# Paraneoplastic Syndrome Presenting Combined Central and Peripheral Demyelination Associated with Anti-CV2/CRMP5 and Anti-NF186 Antibodies: A Case Report

**DOI:** 10.3390/brainsci13030374

**Published:** 2023-02-21

**Authors:** Bingyou Liu, Lei Zhou, Yongsheng Zheng, Chong Sun, Jie Lin

**Affiliations:** Department of Neurology, Huashan Hospital, Fudan University, 12 Middle Wulumuqi Road, Jingan, Shanghai 200040, China

**Keywords:** paraneoplastic syndrome, combined central and peripheral demyelination, anti-CV2/CRMP5, anti-NF186

## Abstract

The anti-CV2/CRMP5 antibody is a well-characterized biomarker of paraneoplastic neurological syndrome. The anti-NF186 antibody is a recently discovered antibody associated with central or peripheral demyelination. The co-occurrence of these two antibodies has not been reported. Herein, we report a case with anti-CV2/CRMP5 and anti-NF186 antibodies in a 57-year-old male presenting with progressive numbness and weakness in his four limbs. At first admission, the spinal cord MRI showed a cervical cord demyelinating lesion and electrophysiological examination showed a mixed demyelinating and axonal polyneuropathy. Anti-CV2/CRMP5 and anti-NF186 antibodies were both detected in his serum. Initially, the patient showed a positive response to IVIG and glucocorticoid treatment. However, the syndrome relapsed and mass lesions in lung and mediastinum were detected at second admission. This time the anti-NF186 antibody was not detected but the anti-CV2/CRMP5 antibody was still present. IVIG and glucocorticoid treatment was no longer effective. This case illustrated that paraneoplastic syndrome should be considered when diagnosing patients with central and peripheral demyelination, and that the anti-NF186 antibody may help distinguish a subset of patients who can benefit from immunomodulatory treatments.

## 1. Introduction

The demyelination disorder involving both the central nervous system (CNS) and the peripheral nervous system (PNS) is named combined central and peripheral demyelination (CCPD) [1]. The anti-CV2/CRMP5 antibody was previously identified as a biomarker of paraneoplastic neurological syndrome, during which CNS or PNS involvement was reported [2,3]. Neurofascin 186 (NF186) is a cell adhesion molecule located on axons of the central and peripheral nervous system. Recent studies indicated that the presence of antibodies against NF186 may indicate acquired peripheral neuropathy or CNS demyelination [4,5,6,7]. However, NF186 was not detected in the CCPD cases. Herein, we reported a CCPD case positive for anti-CV2/CRMP5 and anti-NF186 antibodies.

## 2. Case Report

A 57-year-old male was admitted with progressive numbness and weakness of his four limbs for seven months. The numbness of his distal lower limbs appeared in initiation and gradually elevated to his thighs after three months. One month later, his lower limb weakness appeared, and he was unable to walk independently. Considering immune-mediated neuropathy, intravenous glucocorticoid was administrated, but symptoms worsened progressively, and his distal upper limbs were involved. Micturition and defecation disturbances appeared simultaneously. He was a non-smoker with normal growth and development history. He was retired and had not been exposed to toxins. No similar or related symptoms were detected in his family. A 15 kg loss of body weight had been observed since the emergence of numbness.

Neurological examination demonstrated no cranial nerve involvement. Muscle strength was graded 3–4 (4 proximal and 3 distal) in the upper limbs and graded 1 in the lower limbs using the medical research council (MRC) score. Superficial and vibration sensations decreased in all limbs and the distal involvement was more prominent. Sensory disturbance was found at T4 in the thoracic spine. All extremities had areflexia. The Babinski sign was bilaterally positive. Finger-to-nose alternating movements were normal.

**Figure 1:**
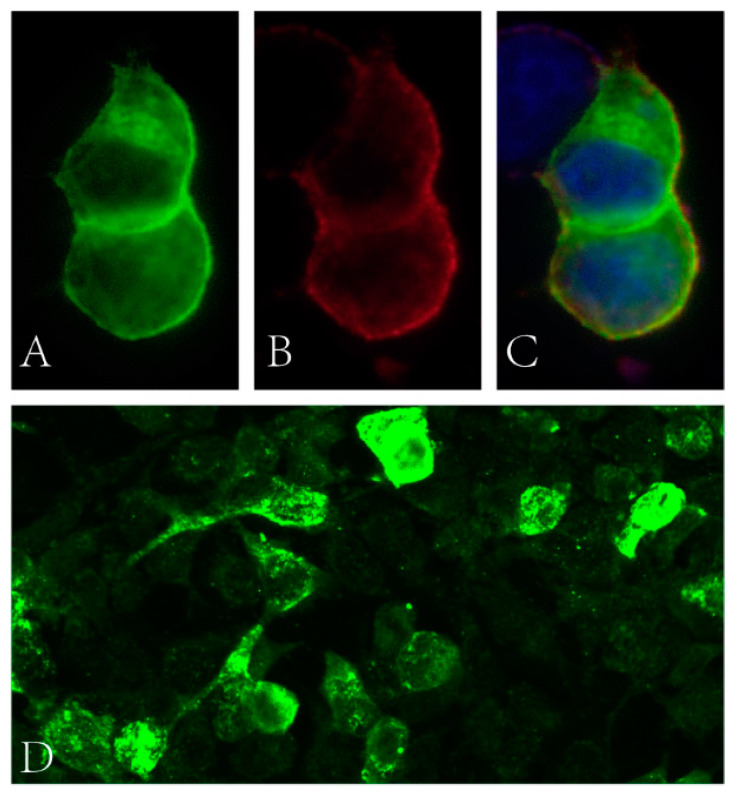
(**A**–**C**) Binding of patient’s serum to HEK cells transfected with NF186 (green fluorescence: NF186 protein; red fluorescence: human IgG; blue fluorescence: nucleus). (**D**) Binding of patient’s serum to HEK cells transfected with CV2 (green fluorescence: human IgG).

**Figure 2:**
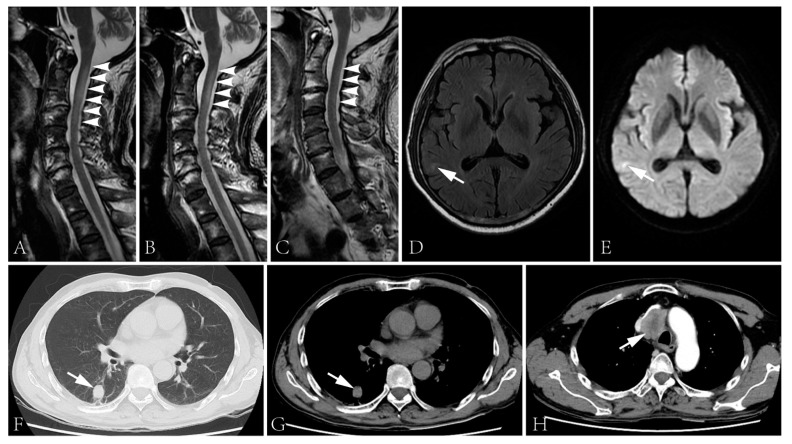
(**A**–**C**) Spinal cord MRI showed T2 signal abnormality in the cervical cord at first admission (**A**), two days after the initiation of intravenous glucocorticoid (**B**) and second admission (**C**). The spinal cord lesion shortened after the administration of intravenous glucocorticoid and was merely visible during the second admission. (**D**,**E**) T2 flair (**D**) and DWI (**E**) images of the brain showed right temporal lobe hyperintensity. (**F**–**H**) The CT scan showed a lung lesion (**F**,**G**) and a mass lesion with enhancement in the superior mediastinum (**H**).

Blood glucose, HbA1c, folic acid and vitamin B12 values were within their normal range. HIV and syphilis screening results were negative. Immunoglobulin light chain and monoclonal immunoglobulin was not detected in blood and urine samples. ANA was positive (1:100). Anti-dsDNA, ENA and ANCA antibodies were all negative. T-SPOT.TB, G and GM tests were all negative. The CSF protein level was 928 mg/L (normal range: 120–600 mg/L) with a slightly elevated WBC count (14 × 10^6^/L, normal range: 0–8 × 10^6^/L). The IgG index analysis demonstrated mild blood–brain-barrier impairment. CSF-restricted oligoclonal bands were detected. CSF cytology demonstrated no malignancy. Anti-NF186 and anti-CV2/CRMP5 antibodies were detected using cell-based assays (Figure 1, anti-NF186 1:32; Anti-CV2/CRMP5 1:100), which were further verified by immunodot blot assay. Anti-AQP4, anti-MOG, anti-ganglioside and other paranodal and paraneoplastic antibodies were not detected. Spinal cord MRI examination showed a cervical cord lesion (Figure 2A). The brain and brachial plexus MRI results were normal. The electrophysiological exam showed a mixed demyelinating and axonal polyneuropathy (see Table S1 in Supplementary Material). PET-CT results revealed a small pulmonary nodule and a mediastinal lymph node with increased FDG metabolism.

Intravenous immunoglobulin was administrated (0.4 g/kg/d × 5d), followed by intravenous methylprednisolone (160 mg/d × 5d, tempered to 80 mg/d × 5d). The numbness of his four limbs and weakness of his upper limbs were alleviated post-treatment. The sensory disturbance level descended to T10. The muscle strength increased to 4+ in his proximal upper limbs and 4 in distal upper limbs. Moreover, micturition and defecation disturbances improved. The lesion in the cervical cord improved (Figure 2B). However, the biopsy of the lung lesion and lymph node were rejected. He was discharged with oral prednisone, 40 mg/d.

The patient gradually regained full control of micturition and defecation and was able to walk around the bed with aids. However, his symptoms recurred five months after discharge. After an upper respiratory tract infection, the numbness and weakness deteriorated quickly. The acute relapse rendered him completely bedbound in one week. His general status declined rapidly with prominent weight loss.

The patient was admitted again three days after being bedbound. His muscle strength was graded 0 in the lower limbs, and the sensory disturbance elevated to T2 level. Other neurological examinations were similar to the first admission. Epileptic attacks were not observed. The anti-NF186 antibody was undetectable but the anti-CV2/CRMP5 antibody was still detected. The CSF protein level was 497 mg/L (normal range: 120–600 mg/L) with a normal cell count. MRI examination revealed a cervical cord lesion and a brain lesion of the right temporal lobe without prominent enhancement (Figure 2C–E). No further evidence of thromboembolic stroke or metastasis was detected. The pulmonary nodule prominently enlarged and a mass lesion with enhancement was observed in his superior mediastinum by CT scans (Figure 2F–H).

Intravenous immunoglobulin and glucocorticoid were administrated again. The rapid progression of the symptoms was retarded, but without full recovery. The biopsy of lung lesion was rejected. The patient died one year later.

## 3. Discussion

Herein, we demonstrate the presence of anti-CV2/CRMP5 and anti-NF186 antibodies in a patient with peripheral and central involvements. CCPD has been rarely reported. It is a clinical diagnosis used to describe demyelinating conditions affecting both the CNS and PNS. Several antibodies were detected in CCPD patients [8,9,10,11]. However, the relationship between CCPD and paraneoplastic neurological syndrome has seldom been discussed. The anti-CV2/CRMP5 antibody was a well-characterized antibody justifying the diagnosis of paraneoplastic syndrome, among which polyneuropathy with CNS involvement was previously reported [12]. Although the pathogenicity of anti-CV2/CRMP5 antibody has not been well characterized, the presence of anti-CV2/CRMP5 antibody may indicate an autoimmune mechanism of disease progression. From the angle of paraneoplastic syndrome, we suppose the diagnosis of the current case may be included in this disease spectrum from a probable status to definite paraneoplastic syndrome utilizing the diagnosis criteria: sensory-motor polyneuropathy plus CNS involvement as an intermediate-risk phenotype, score 2; anti-CV2/CRMP5 antibody as a high-risk antibody, score 3; carcinoma, score 1 or 4 [3]. However, the presence of the anti-NF186 antibody was intriguing. The anti-NF186 antibody was previously reported in patients with either demyelinating/axonal peripheral neuropathy or CNS demyelination [4,7]. A previous study revealed some patients with paraneoplastic syndrome had antibodies against DRG neurons, motor neurons, or Schwann cells, though anti-NF186 antibody was not detected [13]. In the current case, this patient showed prominent response to immunomodulatory treatments without carcinoma management, which was rare in previous reports describing paraneoplastic syndrome. The nerve conduction study revealed prominent demyelinating features, which was not observed in previous studies [14,15]. The variation of the clinical phenotype and examination results from a “classical” paraneoplastic syndrome may be caused by the NF186 antibody. Located on the axon, NF186 is a cell surface protein which is essential for the assembly and normal function of the Ranvier node [16,17]. Antibodies against two major neurofascin isoforms were detected in patients with CNS and/or PNS symptoms [11,18,19]. In addition, the anti-pan neurofascin antibody was also reported in a small subset of patients with fulminant neuropathy [20,21]. An antibody against the glial neurofascin isoform, NF155, was predominantly of IgG4 subtype, which lacks the ability to activate a conventional complement-mediated or cellular immune response. A recent study revealed that anti-NF155 IgG4 was pathogenic because it rendered NF155 degeneration directly, whereas the anti-NF186 antibody may be more widely distributed in different antibody isotypes or IgG subtypes, and it may be pathogenic in a complement dependent manner, similar to the pathogenic mechanism of anti-pan-neurofascin antibody [6,22]. This may also be the underlying mechanism of the effective glucocorticoids and IVIG treatment as shown by both this study and other studies. The diminishment of anti-NF186 after treatment further indicated the effect of anti-NF186 in disease progression. However, the detection of probable primary carcinoma and the poor prognosis justified the presence of the anti-CV2/CRMP5 antibody, which suggested that the paraneoplastic syndrome may play an essential role in disease progression. Co-occurrence of the paraneoplastic antibody and other antibodies related to a neurological syndrome has seldom been reported. Bien et. al. reported a case diagnosed with paraneoplastic encephalitis along with antibodies against dipeptidyl-peptidase-like protein-6 and aquaporin-4 [23]. However, the case may be easier to define because the patient presented with a more “classical” phenotype of paraneoplastic syndrome rather than a combination of paraneoplastic syndrome and neuromyelitis optica spectrum disorder. Furthermore, the two antibodies detected were both defined as low-risk paraneoplastic antibodies in the new diagnostic criteria. In our case, the relationship between anti-NF186 antibody and cancer was not well characterized. Patients with anti-NF186 antibody were reported to be associated with concomitant autoimmune disorders, but no patient presented with evidence of tumor of a paraneoplastic origin. In addition, the clinical phenotype in our case was an atypical paraneoplastic syndrome. Thus, we also gave a diagnosis of CCPD, given the clinical manifestation and radiological evidence. Whether these two antibodies originated from the same site and whether they were both related to tumor immunology remains to be further elucidated.

One limitation of the present study is that the diagnosis of malignancy was not verified by pathology examination. However, as the patient has passed away, it is not possible to obtain a tissue specimen retrospectively. Considering the continuous presence of the paraneoplastic antibody, the prominently enlarged lung and lymph node lesions and the results of PET-CT, we hypothesize that lung cancer is likely to be a correct diagnosis for this patient. As paraneoplastic syndrome is an exclusive diagnosis, several possible differential diagnoses were considered in the procedure of diagnosis in our case. We did not detect further evidence of tuberculosis, sarcoidosis, fungi infection and other commonly considered diagnoses. This patient was a non-smoker, the position of the mass lesion did not show prominent association with trachea and bronchi and there was rapid disease progression. The anti-CV2/CRMP5 antibody was also reported to be commonly associated with small-cell lung carcinoma and adenocarcinomas. Thus, we can only make a speculation based on the currently available data that the primary malignancy was likely to be small-cell lung carcinoma or pulmonary adenocarcinoma. Further evidence is required to verify our report.

## 4. Conclusions

Although an immune-mediated disease was commonly considered when diagnosing a patient with CNS and PNS “demyelination”, paraneoplastic syndrome should not be ruled out. In addition to antibodies against neuron and Schwann cells that may be detected in patients with paraneoplastic syndrome, the anti-NF186 antibody may play a pathogenic role. The screening of anti-NF186 antibody in patients diagnosed with paraneoplastic syndrome may help identify the subset of patients who can benefit from immunomodulatory treatments. However, the treatment of carcinoma is still fundamental for long-term survival. Further investigations are required to elucidate the relationship between anti-NF186 antibody and paraneoplastic syndrome.

## Data Availability

The data presented in this study are available on request from the corresponding author.

## Supplementary Materials

The following supporting information can be downloaded at: https://www.mdpi.com/article/10.3390/brainsci13030374/s1, Table S1: Electrophysiological results.
