# Supplementary material for: Paraneoplastic Syndrome Presenting Combined Central and Peripheral Demyelination Associated with Anti-CV2/CRMP5 and Anti-NF186 Antibodies: A Case Report
Source: Brain Sci. 2023 Feb 21;13(3):374. doi: 10.3390/brainsci13030374 (PMC10046875; doi:10.3390/brainsci13030374)
Supplement: Supplementary file 1 [file brainsci-13-00374-s001.zip › brainsci-2089206-supplementary.pdf]

Table S1. Electrophysiological results.

	Left	Right	Normal Value
**Median nerve**			
MCV (m/s)	45.5	46.3	≥50.0
Distal CMAP (mV)	4.1	3.6	≥4.8
Proximal CMAP (mV)	4.0	3.5	-
DML (ms)	4.77	4.84	≤4.2
F wave latency (ms)	34.5	36.0	≤30.1
SCV (m/s)	50.9	43.9	≥50.8
SNAP (μV)	8.6	6.8	≥8.0
**Ulnar nerve**			
MCV (m/s)	ND	49.5	≥50.0
Distal CMAP (mV)	8.5	6.5	≥5.5
Proximal CMAP (mV)	ND	5.9	-
DML (ms)	3.33	4.32	≤3.1
F wave latency (ms)	37.8	38.3	≤33.0
SCV (m/s)	-	40.0	≥50.6
SNAP (μV)	NP	3.0	≥5.0
**Tibial nerve**			
MCV (m/s)	ND	39.5	≥39.4
Distal CMAP (mV)	ND	3.7	≥5.0
Proximal CMAP (mV)	ND	2.9	-
DML (ms)	ND	4.87	≤5.8
F wave latency (ms)	ND	66.9	≤60.2
**Superficial peroneal nerve**			
SCV (m/s)	ND	34.4	≥41.3
SNAP (μV)	ND	0.93	≥4.0
**Sural nerve**			
SCV (m/s)	ND	37.7	≥41.9
SNAP (μV)	ND	2.3	≥7.0

ND: not done; NP: no potential
